# The Graphene Quantum Dots Gated Nanoplatform for Photothermal-Enhanced Synergetic Tumor Therapy

**DOI:** 10.3390/molecules29030615

**Published:** 2024-01-27

**Authors:** Lipin Wang, Wenbao Wang, Yufang Wang, Wenli Tao, Tingxing Hou, Defu Cai, Likun Liu, Chang Liu, Ke Jiang, Jiayin Lin, Yujing Zhang, Wenquan Zhu, Cuiyan Han

**Affiliations:** 1 School of Pharmacy, Qiqihar Medical University, Qiqihar 161006, China; wlp433477@163.com (L.W.); wangwenbao0824@163.com (W.W.); wangyufang1998@163.com (Y.W.); twl9115@163.com (W.T.); houtingxing@163.com (T.H.); qdliuchang@163.com (C.L.); 17835218902@163.com (Y.Z.); 2 Institute of Medicine and Drug Research, Qiqihar Medical University, Qiqihar 161006, China; cai@qmu.edu.cn (D.C.); llk_2008@126.com (L.L.); 3 Qiqihar Center for Disease Control and Prevention, Qiqihar 161006, China; jk95177@163.com; 4 College of Discipline Inspection and Supervision, Qiqihar Medical University, Qiqihar 161006, China; ljy020223@126.com

**Keywords:** mesoporous carbon nanoparticles, graphene quantum dots, multi-stimuli responsive release, enhanced permeability, synergistic therapy

## Abstract

Currently, the obvious side effects of anti-tumor drugs, premature drug release, and low tumor penetration of nanoparticles have largely reduced the therapeutic effects of chemotherapy. A drug delivery vehicle (MCN-SS-GQDs) was designed innovatively. For this, the mesoporous carbon nanoparticles (MCN) with the capabilities of superior photothermal conversion efficiency and high loading efficiency were used as the skeleton structure, and graphene quantum dots (GQDs) were gated on the mesopores via disulfide bonds. The doxorubicin (DOX) was used to evaluate the pH-, GSH-, and NIR-responsive release performances of DOX/MCN-SS-GQDs. The disulfide bonds of MCN-SS-GQDs can be ruptured under high glutathione concentration in the tumor microenvironment, inducing the responsive release of DOX and the detachment of GQDs. The local temperature of a tumor increases significantly through the photothermal conversion of double carbon materials (MCN and GQDs) under near-infrared light irradiation. Local hyperthermia can promote tumor cell apoptosis, accelerate the release of drugs, and increase the sensitivity of tumor cells to chemotherapy, thus increasing treatment effect. At the same time, the detached GQDs can take advantage of their extremely small size (5–10 nm) to penetrate deeply into tumor tissues, solving the problem of low permeability of traditional nanoparticles. By utilizing the photothermal properties of GQDs, synergistic photothermal conversion between GQDs and MCN was realized for the purpose of synergistic photothermal treatment of superficial and deep tumor tissues.

## 1. Introduction

Cancer has become a serious hazard to human health. Accordingly, a variety of powerful means to treat tumors have been developed, including surgical excision, chemotherapy, radiation, etc. [1,2]. Among them, chemotherapy is becoming a popular cancer treatment option. Nano-drug delivery systems have developed quickly, but due to the lack of tumor-specific targeting capability and multi-drug resistance, conventional chemotherapy still has certain shortcomings, such as the high toxicity of anticancer drug and ineffective individual chemotherapy.

Photothermal therapy (PTT) is a type of physical therapy that destroys tumor cells by converting light into cytotoxic heat energy [3]. PTT has received a great deal of interest in cancer treatment, since it is a non-invasive, efficient method with few side effects [4]. Near-infrared (NIR) light has a wavelength of 700–1100 nm, which is less harmful to the human body due to its deep penetration. It is absorbed by nanocomposite materials and transformed into poisonous hyperthermia in tumors [5]. The resulting high temperature promotes the release of chemotherapeutic drugs loaded in the carrier, promotes the uptake of nanocomposites by cancer cells, and sensitizes tumor cells to chemotherapy drugs [6,7], thus greatly reducing dose requirements and side effects. Therefore, the effects of PTT chemotherapy can be superior to those of monotherapy [8].

Numerous functional PTT materials, including metal oxides or sulfides, MOF-based materials [9], precious metal materials, and carbon-based nanomaterials [10,11,12,13,14], have been created as carriers for NIR absorption agents due to the fast development of PTT. Mesoporous carbon nanoparticles (MCN), one of these PTT adsorbents, have drawn a significant amount of interest owing to their excellent properties, including high surface area and porosity, tailorable mesopore size, and easily modified surface. Except for the aforementioned characteristics, the excellent photothermal conversion efficiency and π–π stack of MCN can increase drug loading and achieve controlled drug release [15], which makes MCNs extraordinary nanocarriers for combined chemotherapy and PTT.

However, there is still a need for improvement in the use of MCN-based chem-PTT drug delivery systems. The drug′s unwanted release prior to reaching the target site may also have substantial toxicity and negative effects on healthy cells, thus leading to unfavorable outcomes. Fortunately, drug delivery systems in response to multifarious stimuli have advanced significantly in recent years. The release of drugs in specific target tissues can be triggered by convenient switching effects brought on by specific stimuli such as intracellular high-concentration glutathione (GSH) [16], lysosomal pH [17], enzymes [18], heat [3], light [14], and magnetic stimuli [19]. Redox potential is one of these stimuli that frequently stimulates intracellularly at the tumor site [20]. The glutathione (GSH) concentration has been found to be significantly different between intracellular (10 mM) and extracellular (2 μM) environments [20]. The GSH levels in most tumor cells are typically about four times greater than in normal cells. Furthermore, factors such as a dense extracellular matrix, increased interstitial fluid pressure, and vascular disorders in solid tumors lead to limited tumor penetration, which seriously hinders targeted drug delivery [21,22]. It is widely accepted that higher diffusional capabilities are present in nanoparticles with smaller particle sizes [23,24], and smaller NPs are conducive to tumor penetration. In this case, nanoparticles with variable particle dimensions have been created that have comparatively large initial dimensions, can extend in vivo time, and can be quickly broken down into small particles to enhance diffusion within the tumor [25]. The large initial dimensions can also prevent the rapid clearance of small particles from blood circulation [25]. Therefore, tiny NPs with exciting photothermal effects are gated on the MCN surface to prevent the unintentional release of chemotherapeutic drugs. The resulting dissociated NPs can penetrate deeply into the interior of the tumor to produce hyperpyrexia, enabling effective chemotherapy–PTT therapy at both the shallow and deep sites in targeted tumors.

Due to their features, such as strong luminescence, high photostability, good hydrophilicity, ease of synthesis and modification, and good biocompatibility, carbon dots (CDs), a new form of fluorescent nanoparticle, have received significant interest [26]. Most importantly, the size of controllable CDs makes them gatekeepers to effectively prevent drug leakage [27,28]. Graphene quantum dots (GQDs), a type of carbon dot, has been extensively used for drug delivery and bioimaging. Therefore, GQDs with outstanding photothermal conversion ability are required in order to realize PTT in deeper tumors by utilizing the photothermal effect of GQDs [29]. Furthermore, the released GQDs with diameters between 3 and 10 nm unquestionably have a greater ability to penetrate and are capable of inducing PTT effects in deep tumor tissues.

**Figure 1:**
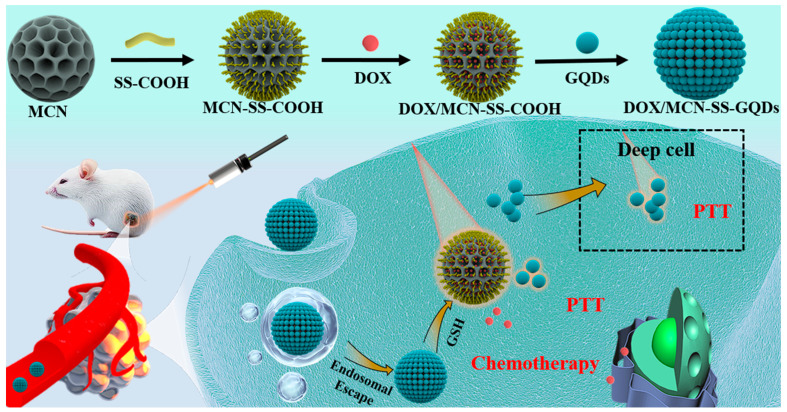
Preparation of intelligent DOX/MCN-SS-GQDs with photothermal enhancement synergy for deep tumor treatment.

In this study, MCN was used as a NIR-absorption drug carrier to achieve synergistic PTT and chemotherapy. With the goal of preventing the premature drug release, small fluorescent GQDs with superior tumor-penetrating capacity and photothermal properties were gated at the opening of MCN to develop an MCN-SS-GQDs delivery system using disulfide bonds, as shown in Figure 1. Doxorubicin (DOX) was selected to investigate the drug-loading capacity, stimulus-reactive release properties, and synergistic effects of thermochemotherapy. Once DOX/MCN-SS-GQDs is transported to the tumor and uptaken by tumor cells, the disulfide bonds are broken by GSH, resulting in the release of DOX within the tumor region. Detached GQDs have the ability to penetrate deep into the tumor, produce cytotoxic heat to boost the PTT effect when exposed to NIR radiation, and ultimately further accelerate drug release and improve the tumor′s susceptibility to chemotherapy drugs. In summary, simple and versatile DOX/MCN-SS-GQDs can achieve stimuli-triggered release, high loading capacity, improved tumor penetration, and a visualized delivery process, thus realizing synergistic chemo-photothermal therapy.

## 2. Results and Discussion

### 2.1. Characterization of GQDs and af-GQDs

**Figure 2:**
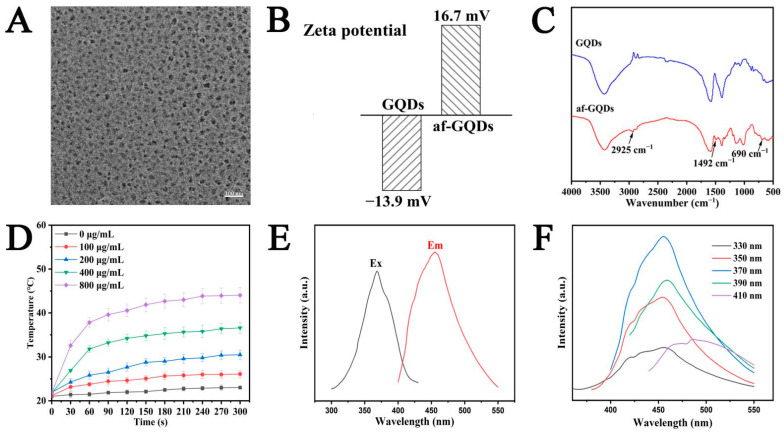
(**A**) TEM image of the GQDs; (**B**) Zeta potential results of GQDs and af-GQDs; (**C**) FT-IR spectra of GQDs and af-GQDs; (**D**) the concentration-dependent photothermal temperature rise curves of GQDs; (**E**) ultraviolet-visible absorption, excitation, and emission spectra of GQDs, illustrated by photographs of GQDs in bright field of view (left) and ultraviolet light (right); (**F**) PL spectra of GQDs under different excitation conditions.

GQDs were provided by Nanjing XFNANO Materials Tech Co., Ltd. (Nanjing, China). In order to covalently graft GQDs onto the surface of MCN as a gated switch, the aminated GQDs (af-GQDs) were prepared. The sizes and morphologies of the GQDs were observed using TEM. In Figure 2A, the GQDs display a monodispersed spherical shape at about 10 nm. The successful preparation of af-GQDs can be characterized by Zeta potential changes and the FT-IR spectrum. It can be seen from Figure 2B that the Zeta potential of af-GQDs reversed from −13.9 mV to a positive charge of 16.7 mV after amination. In addition, the features of surface functional groups of af-GQDs were further evaluated via FT-IR (Figure 2C). Compared to GQDs, a flexural vibration peak of -CH_2_-containing keto bonds at −1492 cm^−1^ was observed. The results showed that the amide bonds were grafted successfully onto the surfaces of af-GQDs.

The fluorescence emission and excitation spectra of GQDs had peak wavelengths of 480 nm and 360 nm, respectively, as illustrated in Figure 2E. The synthesized GQDs were colorless when exposed to natural light and fluoresced brightly blue when exposed to 360 nm ultraviolet light. As shown in Figure 2F, the fluorescence emission spectrum of the GQDs showed fluorescence characteristics independent of the excitation wavelength, and the excitation peak was located at 460 nm. In addition, the thermal performance of GQDs was further studied. The different concentrations of GQDs solution were exposed to NIR illumination for 5 min at a power of 2 W/cm^2^. As shown in Figure 2D, there was no significant temperature change in the water under laser irradiation. In contrast, the GQDs suspensions increased in temperature by about 15 °C at 400 μg/mL, showing a concentration dependence. The temperature rise curves of GQDs under different power densities are shown in Figure S1. The graph clearly illustrates the power-dependent temperature-increasing features of GQDs. To further estimate the photothermal performance of GQDs, the photothermal conversion efficiency (η) values of GQDs were calculated, as shown in Figure S2. The η value of the GQDs was 6.9%, according to the reported literature [30]. In summary, the prepared GQDs had outstanding fluorescence characteristics and eminent photothermal generation efficiency, which provided a strong basis for the employment of NIR-adsorbing agents in the treatment of cancer.

### 2.2. Characterizations of DOX/MCN-SS-GQDs

**Figure 3:**
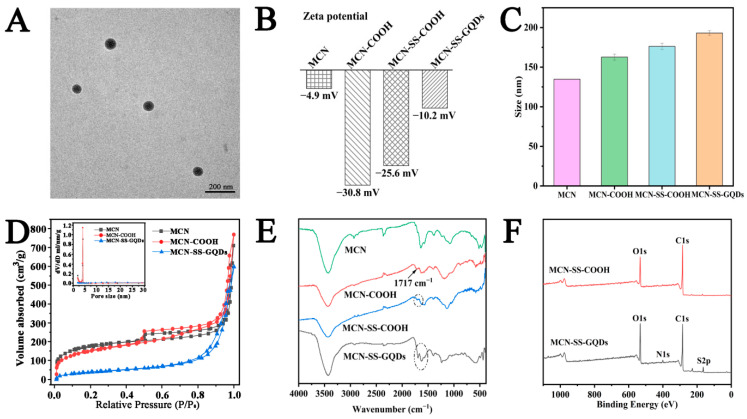
TEM image of (**A**) MCN-COOH; (**B**) Zeta potential; and (**C**) particle size results after each modification; (**D**) nitrogen adsorption isotherms of MCN-COOH, MCN-SS-COOH, and MCN-SS-GQDs; (**E**) FT-IR spectra of each modification stage; (**F**) XPS spectra results.

In order to achieve multi-responsive drug release and photothermal augmentation of synergistic therapy, MCN-SS-GQDs were developed by grafting GQDs as responsive switches onto the mesopores of MCN via cleavable disulfide bonds, as illustrated in Figure 1. The synthetic procedure of MCN-COOH was conducted according to the literature reports [31]. As shown in Figure 3A, MCN-COOH exhibited a homogeneous spherical structure with an average particle size of approximately 120 nm and abundant mesopores, which is suitable for the effective loading and controlled release of chemotherapeutic drugs.

In addition, the effective grafting of GQDs onto the surface of MCN-COOH was confirmed by multifarious methods. The Zeta potential changes modified by every step are displayed in Figure 3B. After the carboxylation modification, the Zeta potential of MCN-COOH decreased from −4.9 mV (MCN) to −30.8 mV. MCN-SS-COOH showed a similar Zeta potential to that of MCN-COOH. After grafting the af-GQDs, the potential of MCN-SS-GQDs drastically dropped to −10.2 mV, indicating the successful grafting of the af-GQDs. As can be seen from Figure 3C, the particle diameter of MCN-SS-GQDs marginally increased after each functionalization. And the final particle size of MCN-SS-GQDs was approximately 180 nm.

The distribution curves, isotherms, and associated parameters of MCN-COOH, MCN-SS-COOH, and MCN-SS-GQDs are exhibited in Figure 3D and Table 1. These results show that the parameters were gradually reduced after the functionalization process, which proved that GQDs had been grafted onto the mesopores of MCN-SS-GQDs. The sizes of the GQDs were similar to the mesopore of the vector, suggesting that GQDs modified at the MCN opening can block the channel and prevent premature drug release. As shown the FT-IR spectra in Figure 3E, it can be seen that, compared with MCN, carboxyl absorption peaks appeared in both MCN-COOH and MCN-SS-COOH at 1717 cm^−1^. After af-GQDs was grafted, the characteristic peaks of 1717 cm^−1^ disappeared. The disappearance of the carboxyl group can provide evidence of the grafting of GQDs on MCN. The existence of GQDs on MCN was also verified using XPS, as shown in Figure 3F. Compared with MCN-SS-COOH, the new peaks of N1s and S2p ascribed to GQDs were observed in the MCN-SS-GQDs, proving the successful grafting of GQDs.

### 2.3. Photothermal Properties of MCN-SS-GQDs

**Figure 4:**
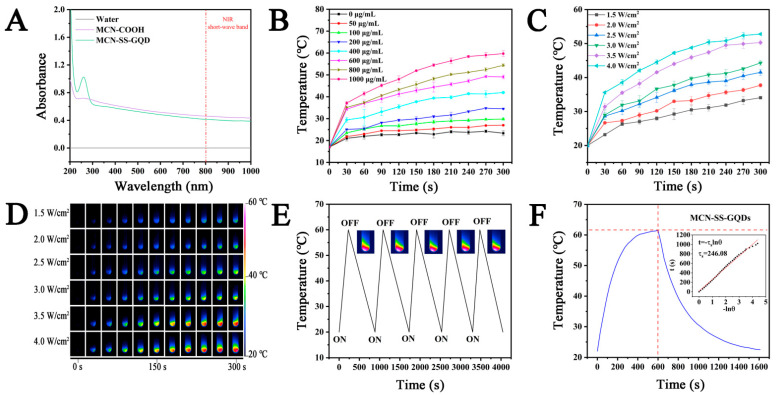
(**A**) UV-Vis-NIR spectra of different samples; (**B**) photothermal rise of series concentration MCN-SS-GQDs suspension at 3.5 W/cm^2^ power; (**C**) line diagram of photothermal temperature rise of series power MCN-SS-GQDs suspension at 600 μg/mL concentration; (**D**) photothermal heating curve of series power MCN-SS-GQDs suspension at 400 μg/mL concentration; (**E**) photothermal stability of MCN-SS-GQDs; (**F**) primary temperature-cooling cycle of nanoparticles (linear time data to -lnθ from the cooling phase of the MCN-SS-GQDs).

For photothermal agents, the capacity for photothermal conversion is crucial. Both MCN-COOH and MCN-SS-GQDs, as seen in Figure 4A, displayed high light absorption in the NIR short-wave range, proving the potential of NIR photothermal conversion. As shown in Figure 4B, MCN-SS-GQDs exhibited good photothermal heating characteristics under NIR light irradiation, while the temperature of the blank control group hardly changed. Under the power condition of 3.5 W/cm^2^, with the increased concentration of the MCN-SS-GQDs suspension, the temperature increased rapidly. The MCN-SS-GQDs temperature rose by about 60 °C at 1 mg/mL within 5 min. The power-dependent photothermal heating curves of MCN-SS-GQDs are shown in Figure 4C. The temperature of MCN-SS-GQDs rose by about 50 °C at a sample concentration of 600 μg/mL within 5 min at 4 W/cm^2^. In order to demonstrate the heating generation ability of MCN-SS-GQDs, we used a NIR thermal imager to capture real-time thermal images of the nanoparticles, as shown in Figure 4D. In summary, the photothermal heating results of MCN-SS-GQDs showed obvious time-dependence, concentration-dependence, and power-dependence properties. The photothermal stability result of MCN-SS-GQDs is shown in Figure 4E. It can be seen that the MCN-SS-GQDs still showed relatively stable photothermal heating performances after five cycles of cooling and heating under NIR light irradiation. As shown in Figure 4F, the MCN-SS-GQDs suspension (50 μg/mL) was irradiated under NIR light and then allowed to cool naturally. The inset in Figure 4F represents the linear time data between the cooling phases of the photothermal effect curve of the MCN-SS-GQDs. The η value of the MCN-SS-GQDs was 21.4%, according to the method reported in the literature [30].

### 2.4. Multiple-Responsive DOX Release

**Figure 5:**
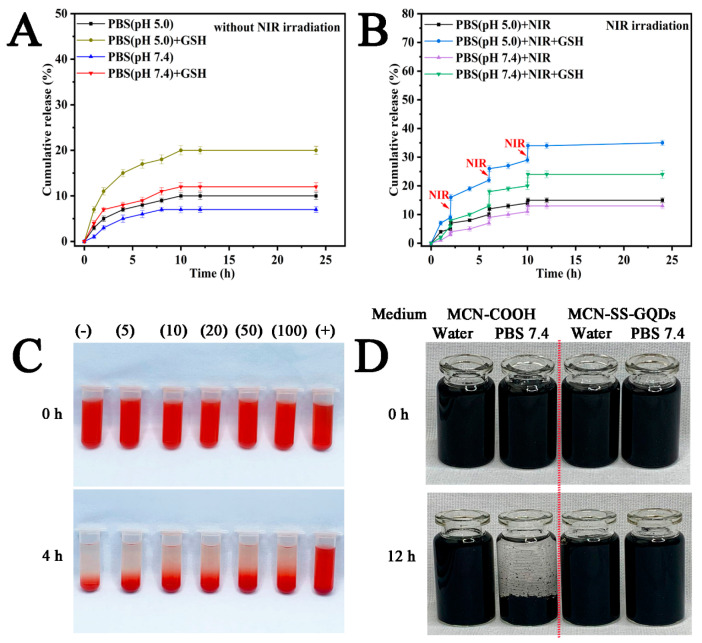
(**A**) Accumulative DOX release of DOX/MCN-SS-GQDs in presence/absence of GSH; (**B**) accumulative DOX release of DOX/MCN-SS-GQDs in pH 5.0 PBS with 10 mM GSH and NIR irradiation at 2.0 W/cm^2^ (*n* = 3); (**C**) hemolysis images of MCN-SS-GQDs at 0 h (top) and 4 h (bottom); (**D**) dispersed images of MCN-COOH and MCN-SS-GQDs in distilled water and PBS.

The LE% and the EE% of DOX in DOX/MCN-SS-GQDs were 29.6% and 41.2%. The high drug loading efficiency was ascribed to the large surface area, order mesopores, and powerful adsorption ability of MCN [32]. To imitate the blood environment and normal tissue, PBS with a pH of 7.4 was employed, and PBS with a pH of 5.0 was utilized to simulate the weakly acidic lysosomal conditions of tumor cells. As illustrated in Figure 5A, the cumulative release of DOX/MCN-SS-GQDs in the pH 5.0 PBS release medium was significantly higher than that in the pH 7.4 PBS release medium, which may have been due to the weak acidic environment, which can weaken the intermolecular interactions between drugs and DOX/MCN-SS-GQDs. The accumulated release of DOX/MCN-SS-GQDs was significantly increased from 10% to 20% in the pH 5.0 PBS group with 10 mM GSH compared to the group without 10 mM GSH. These results show that the DOX/MCN-SS-GQDs′ redox-sensitive release characteristics were caused by the disulfide bonds in the nanoparticles. High GSH caused the breakage of disulfide binds between MCN and GQDs, causing the GQDs to escape from the mesopores of MCN vehicles, thus promoting DOX release from DOX/MCN-SS-GQDs.

Subsequently, 808 nm NIR illumination (2.0 W/cm^2^) was used to irradiate the samples for 3 min at 2 h, 6 h, and 10 h following drug release in order to evaluate the impact of the photothermal effect on drug release. As shown in Figure 5B, drug release was observed with a stepped-like accelerated release performance under the NIR irradiation time points. Finally, the accumulated release of drugs in DOX/MCN-SS-GQDS was 35% in the pH 5.0 PBS release medium under NIR irradiation in the presence of 10 mM GSH, which was significantly increased compared to 15% without GSH or 20% without NIR irradiation. The results showed that DOX/MCN-SS-GQDS had a NIR photothermal effect and redox-responsive release characteristics. To sum up, DOX/MCN-SS-GQDS drug delivery systems showed redox/NIR stimuli-responsive release characteristics.

To further demonstrate the surface separation of GQDs from MCN-SS-GQDs, the MCN-SS-GQDs sample was incubated with 0, 5, and 10 mM GSH for 6 h, and the fluorescence spectra of supernatant GQDs were measured using fluorescence spectra. As shown in Figure S5, no significant fluorescence signal was observed at 0 mM GSH. After separation from MCN-SS-GQDs, the fluorescence intensity of the GQDs increased in a GSH concentration-dependent manner. As shown in Figure S6, without the addition of GSH, the temperature of the MCN-SS-GQDs supernatant did not increase significantly. In contrast, the supernatant of the MCN-SS-GQDs had a significant photothermal effect when incubated with 5 mM GSH or 10 mM GSH. These results suggest that, in the presence of GSH, GQDs can be separated from MCN-SS-GQDs through disulfide bond breaking.

### 2.5. Safety Evaluation of MCN-SS-GQDs

The stability and blood compatibility of nanoparticles are critical for in vivo applications. The biosafety of the prepared nanoparticles, MCN-COOH and MCN-SS-GQDs, were evaluated according to blood compatibility and dispersion stability. As shown in Figure 5C, the hemolysis percentage of MCN-SS-GQDs at 5–100 μg/mL was significantly dose-dependent at 4 h. Even at the high sample concentrations of 50 μg/mL and 100 μg/mL, the hemolysis percentages of MCN-SS-GQDs were still less than 2%, which proved that the MCN-SS-GQDs had good biocompatibility and safety. The reduced hemolysis percentage was attributed to the reduced electrostatic reaction between the MCN-SS-GQDs and erythrocyte membrane proteins after the grafting of GQDs. As shown in Figure 5D, MCN-COOH and MCN-SS-GQDs can exist stably in distilled water for 12 h. In contrast, with the pH 7.4 PBS (simulated physiological condition), MCN-SS-GQDs could remain stable for 12 h, while MCN-COOH precipitated obviously, proving that the biosafety of GQDs-grafted nanoparticles was improved.

### 2.6. Evaluation of Cellular Photothermal Effect

**Figure 6:**
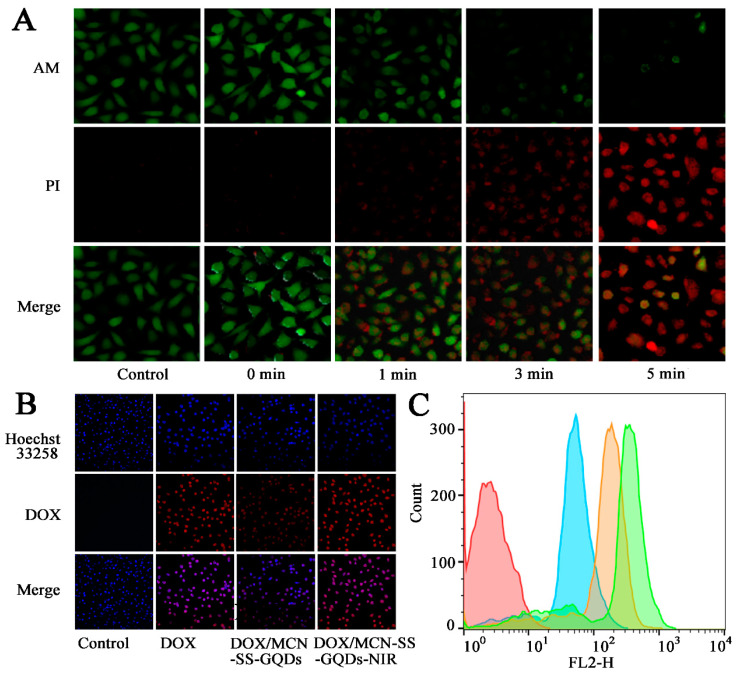
(**A**) Fluorescence images of MCN-SS-GQDs-incubated MCF-7 cells with NIR irradiation (1.0 W/cm^2^), co-stained with calcein-AM and PI for different times. (**B**) CLSM image and (**C**) FCM analysis of MCF-7 cells cultured with DOX, MCN-SS-GQDs and MCN-SS-GQDs + NIR for 4 h. Red—control, Blue—DOX/MCN-SS-GQDs, orange—DOX/MCN-SS-GQDs + NIR, Green—DOX/MCN-SS-GQD + NIR.

In view of the excellent photothermal capability of MCN-SS-GQDs, we further evaluated its photothermal effect at the cellular level. In order to continue investigating the PTT agent effect of MCN-SS-GQD, the survival statuses of cells after NIR irradiation were evaluated using Calcin-AM/PI cellular staining (Figure 6A). Obvious green fluorescence was observed in the control group, indicating the good vitality of the cells. The intracellular red fluorescence (dead cells) was significantly enhanced with the increase in the irradiation time. Within 3 min, a significant amount of red light became visible. With the increase in irradiation duration to 5 min, the intracellular green fluorescence almost disappeared, and the red fluorescence reached the maximum. Therefore, the results of Calcin-AM/PI staining show that MCN-SS-GQDs could completely destroy tumor cells under PTT irradiation because of its good photothermal effect.

### 2.7. Cellular Uptake

CLSM and FCM were employed to assess the uptake of nanoparticles by cells. As illustrated in Figure 6B, in the control group, no red fluorescence signal was observed. The DOX group showed a significant amount of overlap between the red fluorescence signal (free drug) and the blue fluorescence signal (nucleus), demonstrating that the free DOX was mostly found in the nucleus. In contrast, DOX/MCN-SS-GQDs nanoparticles were internalized into the cytoplasm of tumor cells through endocytosis, and the red fluorescence signal of the DOX/MCN-SS-GQDs group was primarily dispersed in the cytoplasm. In addition, the DOX/MCN-SS-GQDs + NIR group showed stronger red fluorescence signals in both the cytoplasm and nucleus, indicating that the photothermal action of NIR can promote the uptake of drugs by cells and the release of intracellular drugs. The drug uptake results measured using FCM were consistent with the CLSM results, as shown in Figure 6C. After receiving NIR laser irradiation, the drug fluorescence value further increased, indicating that the photothermal action of NIR can promote the uptake of drugs by cells and the release of drugs inside the cells. Therefore, the aforementioned findings suggest that DOX/MCN-SS-GQDs could be employed as a delivery nanoplatform for targeted delivery.

### 2.8. Evaluation of Combined Treatment Effect

**Figure 7:**
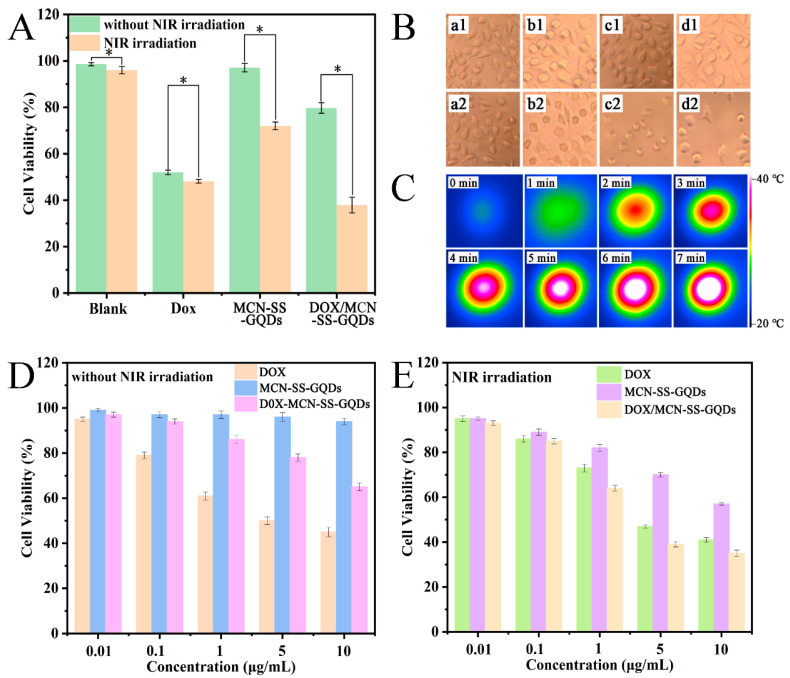
(**A**) Morphology of MCF-7 cells cultured with DOX, MCN-SS-GQDs, and DOX/MCN-SS-GQDs suspensions with or without 3 min NIR irradiation for 24 h, and MCF-7 cell viability of MCF-7 cells cultured in fresh DMEM for 1 h (*, *p* < 0.05). (**B**) Cell morphology of MCF-7 cells cultured with control (a1, a2), DOX (b1, b2), MCN-SS-GQDs (c1, c2), and DOX/MCN-SS-GQDs (d1, d2) suspensions for 24 h with or without 3 min NIR irradiation. (**C**) Infrared thermal images of MCN-SS-GQDs suspension with a concentration of 1 mg/mL, incubated with MCF-7 cells under 808 nm laser irradiation (2.5 W/cm^2^). MCF-7 cells incubated with DOX, MCN-SS-GQDs, and DOX/MCN-SS-GQDs suspensions for 24 h without (**D**) or with (**E**) NIR laser irradiation (2.5 W/cm^2^).

After incubating the cells with different preparations for 1 d, a fluorescence microscope was used to examine the survival state of the cells. As shown in Figure 7B, compared with the blank group (a1 a2), there was almost no significant change for the DOX group in the presence of NIR light irradiation or without it, indicating that DOX had no photothermal effect. In the absence of NIR, MCN-SS-GQDs showed good cell growth when incubated with MCF-7 cells, indicating good biocompatibility. However, some of the MCF-7 cells developed a round-like morphology, and some cellular antennas were missing. For the DOX/MCN-SS-GQDs group, most of the MCF-7 cells developed a round-like morphology under NIR irradiation, indicating the apoptosis or necrosis of the cells. MCF-7 cells were incubated with MCN-SS-GQDs for 1 day before being irradiated with a 808 nm laser, and a thermal imager was employed to measure the temperature. As shown in Figure 7C, with the increase in time, the temperature of the MCF-7 cells gradually increased. The cells incubated with MCN-SS-GQDs showed a significant, time-dependent photothermal heating feature, and the temperatures of the cells exceeded the lethal temperature of the tumor (42 °C) [33]. The experiments demonstrated the effective photothermal conversion of MCN-SS-GQDs.

In addition, the CCK-8 method was employed to quantitatively determine the cell viability of different treatment groups, and the results are shown in Figure 7A. No discernible difference in cytotoxicity was found between the blank group and the DOX group before or after NIR irradiation. However, the cytotoxicity of the nanoplatforms (MCN-SS-GQDs and DOX/MCN-SS-GQDs) increased significantly under laser irradiation. Compared with the single chemotherapy group and the single phototherapy group, the killing effect of the combined treatment group (DOX/MCN-SS-GQDs + NIR) towards MCF-7 cells was significantly increased.

### 2.9. In Vitro Cytotoxicity Evaluation

The cytotoxicity of DOX/MCN-SS-GQDs on MCF-7 cells was evaluated via a CCK-8 assay. To ascertain the impact of chemotherapy–photothermal synergistic therapy, MCF-7 cells were cultured with different samples, and then NIR laser radiation was carried out for the hyperthermia group. The MCF-7 cell survival rate was above 90% after incubation with different concentrations of MCN-SS-GQDs for 24 h, as shown in Figure 7D,E, indicating that the cytotoxicity of MCN-SS-GQDs was low in vitro and that the cytotoxicity of drug-loading nanoparticles came from DOX or the photothermal effect. Concentration-dependent cell inhibition was noticed for the free DOX group. Compared to the free DOX group with the same concentration, DOX exposed to laser radiation had comparable cell viability. And the cell survival rates of the two groups did not change significantly, proving that the cell viability was not significantly inhibited by the NIR laser. For the NIR group, the cell viability of MCF-7 cells incubated with 10 μg/mL MCN-SS-GQDs decreased from 94% to 57%, indicating that hyperthermia induced by NIR light irradiation had obvious toxic effects on the MCF-7 cells. For DOX/MCN-SS-GQDs group with NIR irradiation, the cell viability at 10 μg/mL decreased by 35% from the original 65%. The results show that drug-loaded nanoparticles had good photothermal therapeutic effects on the cancer cells.

Furthermore, the IC_50_ values were calculated as shown in Table 2. As a common index, the combination index (CI) value has been widely used to prove the combined effect of two different therapies [14,34]. The CI value of DOX/MCN-SS-GQDs was 0.60 (less than 0.9), indicating the synergistic effect of PTT and chemotherapy. This can be ascribed to the fact that an increased temperature can accelerate the release of DOX, damage cells, and enhance the sensitivity of chemotherapy towards cancer cells, thus achieving synergistic chemotherapy–photothermal therapy. In conclusion, DOX/MCN-SS-GQDs + NIR in the combined treatment group was able to achieve a synergistic therapeutic effect on cancer through chemical/photothermal therapy.

## 3. Materials and Methods

### 3.1. Reagents

Doxorubicin hydrochloride (DOX, wt > 99.5%) N-hydroxysuccinimide (NHS), glutathione (GSH), N-(3-dimethylaminopropyl)-N-ethylcarbodiimide hydrochloride (EDC), were purchased from Macklin Chemical Inc. (Shanghai, China). RPMI-1640 cell culture, fetal bovine serum (FBS), and penicillin–streptomycin were provided by GIBCO, Invitrogen Co (Carlsbad, CA, USA). Before use, none of the analytical reagents underwent further purification.

### 3.2. Preparation of af-GQDs

A 30.1 mg GQDs sample was dispersed into 20 mL *N*,*N*-dimethylformamide (DMF) via ultrasound, and then 100 μL 3-aminopropyl trimethoxysilane (APTMS) was mixed into the GQDs solution with thorough swirling. The mixture was heated in an oil bath at 120 °C for 20 min. Finally, the reaction liquid was centrifuged to obtain the product of amino-modified GQDs (af-GQDs).

### 3.3. Preparation of DOX/MCN-SS-GQDs

The preparation of MCN-SS-COOH was carried out according to a previously reported work [30]. Then, the MCN-SS-COOH was activated further by EDC and NHS in order to be covalently modified by af-GQDs and stirred overnight. The prepared sample was collected by centrifugation and renamed as MCN-SS-GQDs.

DOX was employed to verify the stimuli-responsive release of MCN-SS-GQDs. A 10.7 mg DOX sample was weighed and dissolved in pH 7.4 PBS via ultrasound, and 10.5 mg MCN-SS-COOH was dispersed in a drug solution via ultrasound. The suspension was further stirred in the dark for 24 h. Then, additional EDC and NHS were added for 1 h, followed by the addition of 5 mg af-GQDs with stirring for 24 h. The sample was then centrifuged to collect the precipitation, and the supernatant was placed into a brown volumetric bottle. The precipitation was cleaned with pH7.4 PBS 3 times. The entire supernatant was measured at 480 nm to calculate the DOX concentration.

The encapsulation efficiency (EE) and loading efficiency (LE) were calculated as follows.

LE% = m_D_/(m_D_ + m_C_) × 100

EE% = m_D_/(m_t_) × 100

where m_D_ is the DOX mass loaded in nanoparticles; m_C_ is the total added carrier mass; and m_t_ is the total DOX mass.

### 3.4. Stimuli-Triggered Release Performance

DOX/MCN-SS-GQD nanopreparations of 20.00 mg were accurately added to 10 mL samples of different release media (pH 5.0, pH 5.0 containing 10 mM GSH, pH 7.4 PBS, pH 7.4 PBS containing 10 mM GSH). These samples were placed onto a shaking table with a constant temperature of 37 °C and a rate of rotation of 100 rpm. At the preset time points, 4 mL of fluid was taken out and the corresponding release medium was supplemented. The absorbances of the drugs were determined at 480 nm, and the cumulative release behaviors of the drugs in each group were determined.

The method used to investigate the NIR-responsive drug release characteristics was roughly the same as the aforementioned drug release method. At 2, 6, and 10 h of drug release, the samples were irradiated using a NIR laser with 2.5 W/cm^2^ power density for 3 min, 4 mL of release fluid was taken and supplemented, and the cumulative drug release was calculated.

### 3.5. In Vitro Evaluation of Photothermal Effect

The concentration-dependent and power-dependent photothermal generation capabilities of MCN-SS-GQDs were determined by irradiating 500 μL samples with an 808 nm laser. A thermal infrared imager was employed to measure the system′s temperature. By irradiating and cooling the samples 5 times, the photothermal stability of the sample was determined. A MCN-SS-GQDs nanodispersion with an ultimate concentration of 1 mg/mL was prepared, and a 500 μL sample was illuminated using a laser at 2.5 W/cm^2^. According to the procedures reported, the photothermal conversion efficiency (PCE) of MCN-SS-GQDs was estimated.

### 3.6. Stability and Hemolysis Test of MCN-SS-GQDs

A hemolysis test was used to detect the blood compatibility of the system. The red blood cells were diluted with saline to prepare a red blood cell suspension with a volume fraction of 2%. Different concentrations of nanocarriers were added to the cell suspension, and the final concentration was between 5 and 100 μg/mL. Saline and distilled water were mixed with the red cell suspension as positive and negative controls, respectively. Hemolysis of the carrier was observed after 4 h. To examine the dispersion and stability of MCN-SS-GQDs, 25 mg nanoparticles (MCN-SS-GQDs and MCN-COOH) were dispersed in pH 7.4 PBS and distilled water for 12 h. Photographs were taken at various time intervals (0 h, 12 h).

### 3.7. Cellular Photothermal Evaluation

The MCF-7 cells were seeded in a 96-well plate overnight. The previous culture medium was replaced with fresh medium with 800 μg/mL of MCN-SS-GQDs, then further cultivated for 24 h. The cells were irradiated using a 2.5 W/cm^2^ 808 nm laser for 5 min. The changes in the photothermal temperature were recorded.

The cells were placed into laser confocal plates, and 10 μg/mL of MCN-SS-GQDs carrier was added and cultured for 2 h. The cells were first cleaned with PBS before being exposed to an 808 nm NIR laser at a power of 1.0 W/cm for 0, 1, 3, and 5 min. The living/dead states of the cells were then observed using a confocal laser scanning microscope (CLSM) after the cells had been stained with Calcein-AM and PI working fluorescent dye.

### 3.8. Cellular Uptake

MCF-7 cells were seeded into 6-well plates for 12 h. DOX/MCN-SS-GQDs and free DOX medium dilution containing 5 μg/mL DOX were added to the plates after cell adhesion growth. The cells in the irradiation group were illuminated with 808 nm light at 1.0 W/cm^2^ for 3 min after 30 min of administration.

After the cells had been incubated for 4 h, a single-cell suspension was prepared with pH 7.4 PBS solution, and the amount of DOX in the cells was determined using flow cytometry (FCM, Calibur, BD, USA). In addition, CLSM was also employed for the purpose of evaluating the uptake performance of MCF-7 cells. The cells cultured in laser confocal plates were incubated with DOX/MCN-SS-GQDs and free DOX (containing the DOX concentration of 5 μg/mL). After 4 h, the slices were stained with Hoechst 33,258 and evaluated by means of CLSM for qualitative evaluation.

### 3.9. In vitro Cytotoxicity Study

The cells were cultured in a 96-well plate, and the 36 holes at the edge of the plate were filled with 100 μL sterile PBS. After the removal of the old culture medium, the DOX, MCN-SS-GQDs, and DOX/MCN-SS-GQDs sample suspensions prepared with DMEM culture medium (equivalent to 0.01, 0.1, 1, 5, and 10 μg/mL DOX, respectively) were added to the MCF-7 cell media for 1 day, and the viabilities of the MCF-7 cells were investigated. In the laser irradiation group, each cell well was irradiated at 808 nm and 2.5 W/cm^2^ for 3 min. Then, CCK-8 (10 μL) was mixed with each well and incubated to determine the viability of the MCF-7 cells. The combined effect of PTT and chemotherapy was evaluated according to the CI value based on the published methods [16,28]. The combination effect was divided into three categories, including indicators of the synergism (CI < 0.9), additive effects (0.9 < CI < 1.1), and antagonism (CI > 1.1).

### 3.10. Characterizations

TEM images (HT7700, Hitachi, Tokyo, Japan) was used to observe the morphology and mesoporous structure of the nanoparticles. The nitrogen adsorption analysis analyzer BSD-1 (Beishide Instrument Technology Co., Ltd., Beijing, China) was used to measure the surface area and pore size distribution of the samples. The particle sizes and Zeta potentials were measured in pH 7.4 PBS with a Zetasizer Nano-ZS90 Nanosizer (Malvern Ltd., Leamington Spa, UK). The fluorescence spectrum of the GQDs was detected using a microplate reader (Tecan Safire 2, Tecan Ltd., Männedorf, Switzerland). The infrared spectrogram of the sample was measured using an Equinox 55 Fourier Transform Infrared Spectrometer (Bruker, Germany).

### 3.11. Statistical Analyses

All the data are presented as mean ± standard deviation (SD). An unpaired, two-tailed *t*-test was used to analyze the significance between groups (* *p* < 0.05).

## 4. Conclusions

In this study, a novel, size-changeable, multi-triggered-release intelligent nanovehicle was developed to achieve a combination of PTT and chemotherapy. The MCN with outstanding photothermal capacity was employed as the delivery vehicle. The nanodrug delivery system (MCN-SS-GQDs) was constructed by grafting the GQDs onto the mesopores of MCN using disulfide bonds. The GQDs were used as the multi-function material, including a PTT agent to enhance the photothermal effect, a switching agent to prevent premature release, and a fluorescent agent to track the release process. The pH/GSH/NIR-responsive release properties of DOX/MCN-SS-GQDs were evaluated using DOX as a model drug. DOX/MCN-SS-GQDs had a high drug loading capacity of 29.6%. DOX/MCN-SS-GQDs remained steady in the circulation, and were rapidly split into DOX/MCN nanoparticles and sufficient GQDs under a high GSH concentration after being internalized by cancer cells. DOX/MCN can be effective in PTT–chemotherapy for superficial tumors. The very small-sized GQDs with enhanced penetration capacity were able to effectively penetrate the deep tumor and carry out photothermal ablation and fluorescence imaging. MCN-SS-GQDs showed an excellent heating effect, a photothermal conversion efficiency of 21.4%, and photothermal stability. DOX/MCN-SS-GQDs showed an obvious photothermal generation effect and increased cellular uptake capacity at the cellular level. In addition, DOX/MCN-SS-GQDs showed significant synergistic chemo-photothermal effects at the cytological level, with a CI of 0.60. This drug delivery platform uses the multi-mode therapeutic method to kill tumor cells at different depths, thus providing a good reference for synergistic tumor treatment.

## Figures and Tables

**Table 1:** The adsorption–desorption parameters of different samples.

Samples	S_BET_ (m^2^/g)	V_t_ (cm^3^/g)	W_BJH_ (nm)
MCN	671.1	1.11	4.1
MCN-COOH	537.2	1.13	4.1
MCN-SS-GQDs	154.3	0.32	-

**Table 2:** IC_50_ value of MCN-SS-GQDs, free DOX, and DOX/MCN-SS-GQDs.

Samples	IC_50_ (μg/mL)	
	Without Laser	NIR Laser
MCN-SS-GQDs	—	10.889
DOX	7.318	7.554
DOX/MCN-SS-GQDs	12.846	5.764

## Data Availability

The data presented in this study are available in article and Supplementary Materials.

## Supplementary Materials

The following supporting information can be downloaded at: https://www.mdpi.com/article/10.3390/molecules29030615/s1. Figure S1. The power-dependent photothermal temperature rise curves of GQDs at 800 μg/mL. Figure S2. Primary temperature-cooling temperature cycle of nanoparticles (linear time data to -lnθ from the cooling phase of the GQDs. Figure S3. otothermal heating curve of series power MCN-SS-GQDs ssuspension at 400 μg/mL concentration. Figure S4. Magnification images from Figure 6B. Figure S5. The fluorescence emission spectra of MCN-SS-GQD ssupernatant under different GSH concentrations. Figure S6. The NIR-induced temperature elevation curves of GQDs at different GSH concentrations (0, 5 and 10 mM of GSH).
