# Supplementary material for: The Graphene Quantum Dots Gated Nanoplatform for Photothermal-Enhanced Synergetic Tumor Therapy
Source: Molecules. 2024 Jan 27;29(3):615. doi: 10.3390/molecules29030615 (PMC10856627; doi:10.3390/molecules29030615)
Supplement: Supplementary file 1 [file molecules-29-00615-s001.zip › molecules-2771811-supplementary.pdf]

Support information

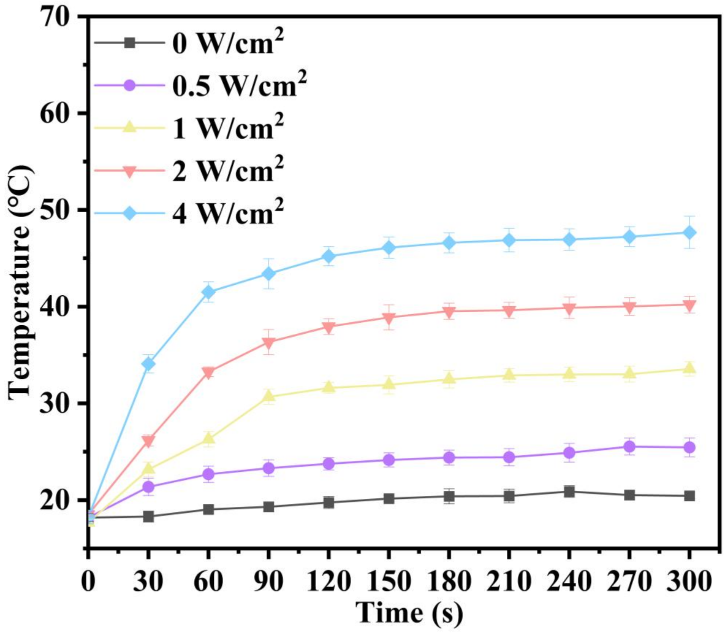

Figure S1 The power-dependent photothermal temperature rise curves of GQDs at 800 μg/mL.

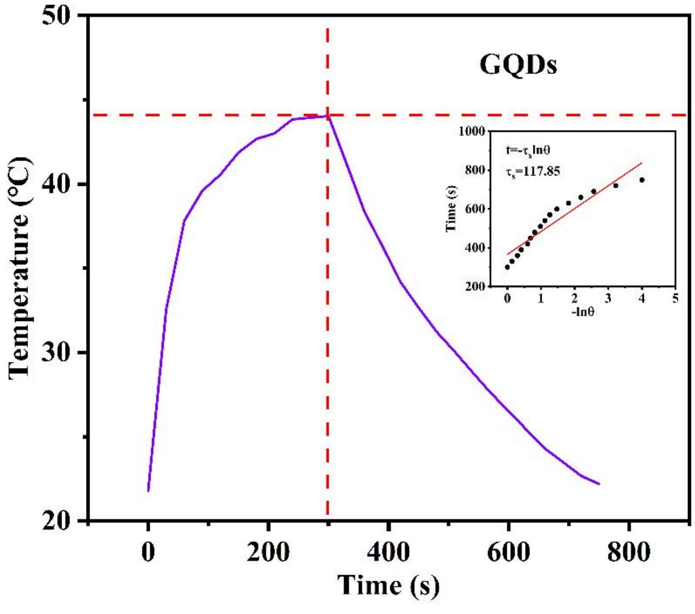

Figure S2 Primary temperature-cooling temperature cycle of nanoparticles (linear time data to -lnθ from the cooling phase of the GQDs

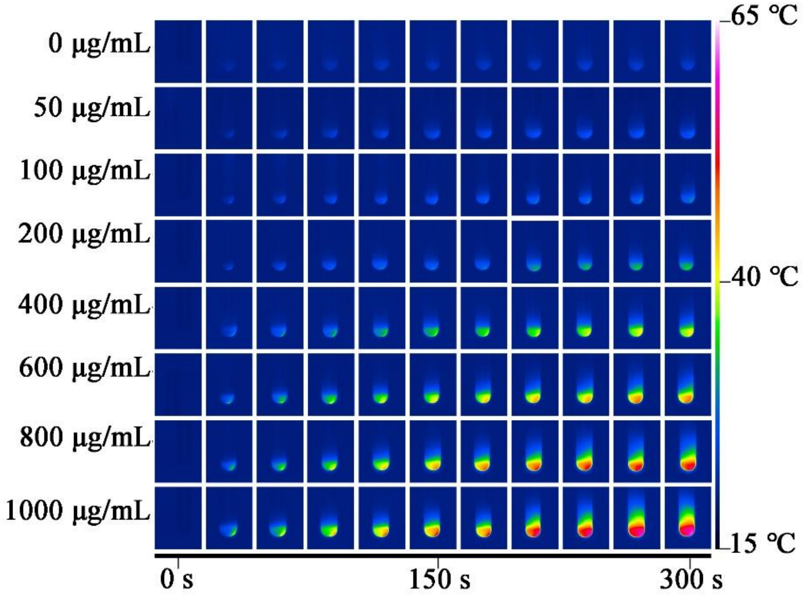

Figure S3 photothermal heating curve of series power MCN-SS-GQDs suspension at 400 μg/mL concentration.

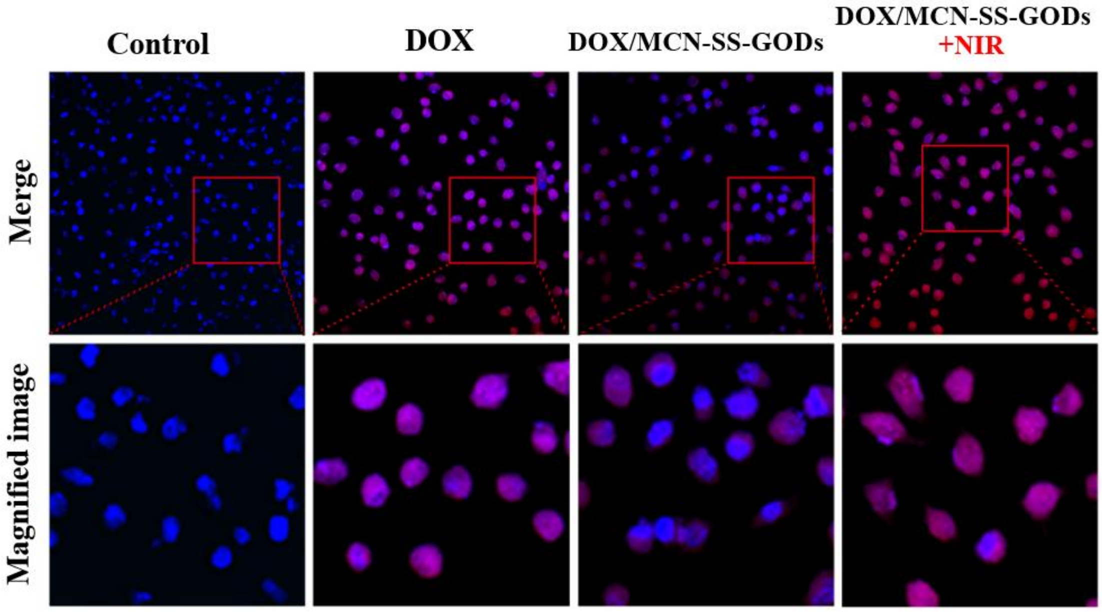

Figure S4 Magnification images from Figure 6 (B).

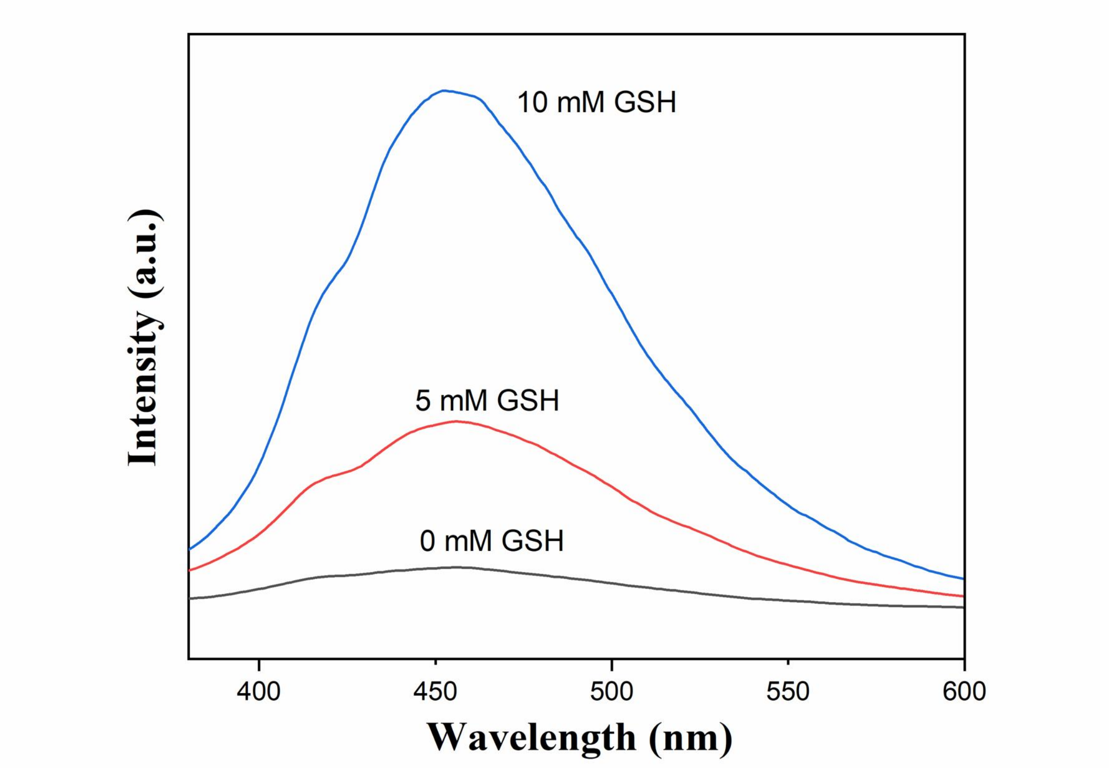

Figure S5 The fluorescence emission spectra of MCN-SS-GQDs supernatant under different GSH concentrations

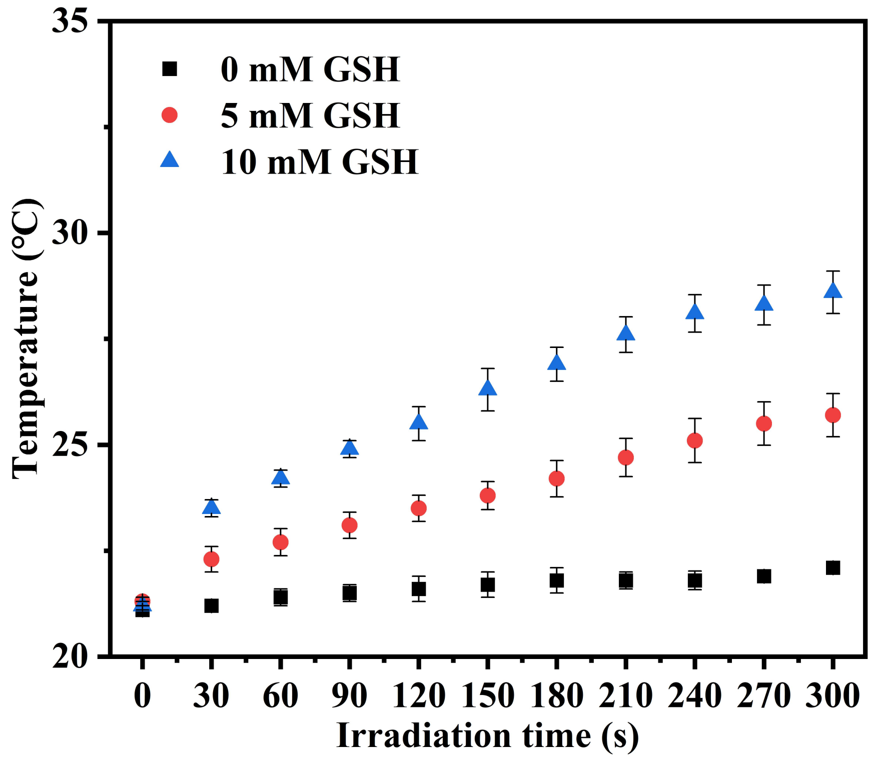

Figure S6 The NIR-induced temperature elevation curves of GQDs at different GSH concentrations (0, 5 and 10 mM of GSH)
